# Study of Chemical Intermediates by Means of ATR-IR Spectroscopy and Hybrid Hard- and Soft-Modelling Multivariate Curve Resolution-Alternating Least Squares 

**DOI:** 10.1155/2017/4595267

**Published:** 2017-03-12

**Authors:** Junxiu Ma, Juan Qi, Xinyu Gao, Chunhua Yan, Tianlong Zhang, Hongsheng Tang, Hua Li

**Affiliations:** ^1^Institute of Analytical Science, College of Chemistry & Materials Science, Northwest University, Xi'an 710069, China; ^2^College of Chemistry and Chemical Engineering, Xi'an Shiyou University, Xi'an 710065, China

## Abstract

3,5-Diamino-1,2,4-triazole (DAT) became a significant energetic materials intermediate, and the study of its reaction mechanism has fundamental significance in chemistry. The aim of this study is to investigate the ability of online attenuated total reflection infrared (ATR-IR) spectroscopy combined with the novel approach of hybrid hard- and soft-modelling multivariate curve resolution-alternating least squares (HS-MCR) analysis to monitor and detect changes in structural properties of compound during 3,5-diamino-1,2,4-triazole (DAT) synthesis processes. The subspace comparison method (SCM) was used to obtain the principal components number, and then the pure IR spectra of each substance were obtained by independent component analysis (ICA) and HS-MCR. The extent of rotation ambiguity was estimated from the band boundaries of feasible solutions calculated using the MCR-BANDS procedure. There were five principal components including two intermediates in the process in the results. The reaction rate constants of DAT formation reaction were also obtained by HS-MCR. HS-MCR was used to analyze spectroscopy data in chemical synthesis process, which not only increase the information domain but also reduce the ambiguities of the obtained results. This study provides the theoretical basis for the optimization of synthesis process and technology of energetic materials and provides a strong technical support of research and development of energy material with extraordinary damage effects.

## 1. Introduction

**Figure 1:**
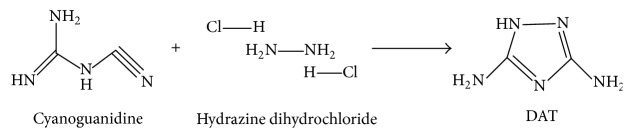
Synthesis route of DAT.

Nitrogen-rich compounds are among the most promising candidates for high energy density materials (HEDM) with the advantages of environmentally benign and high energy density and have been admitted in various areas such as propellants, explosives, and pyrotechnics [1, 2]. 3,5-Diamino-1,2,4-triazole (DAT) as one of the triazole derivatives has been a significant energetic intermediate towards energetic compounds including 5-amino-3-nitro-1,2,4-triazole (ANTA) and 4,6-bis(5-amino-3-nitro-1,2,4-triazol-1-yl)-5-nitropyrimidine (BANTNP) [1, 2]. Moreover, its derivatives have been applied widely in many fields [3–5], such as in dyes [6], cathode catalysts [7, 8] and ligands to transition metals [9–11]. Therefore, there has been growing interest in the synthesis of DAT in recent years [12–14]. The cyanoguanidine and hydrazine dihydrochloride were used to synthesize DAT [14], and achieving a yield of 89% with 99% purity, its synthesis route was shown in Figure 1. The analysis of the structure changes of principal components and the determination of reaction mechanisms and reaction rate constants during synthesis process are of fundamental significance in chemistry and usual practice in process analytical chemistry.

Process analytical technologies (PAT) [15–18] have been employed to design and develop continuously controlled process (by in-line, online, or at-line measurements of the critical intermediate steps and endpoints during the process), so that a predefined quality can be ensured at the end of the manufacturing [15, 16, 18]. To fulfill the real-time analysis of chemical reaction process, it is necessary to use and implement comprehensive PAT tools [16, 19–21] (chemometrics tools, process analyzers, endpoint monitoring tools, and knowledge management tools). Chemometrics enable resolving the complex and large sets of matrix and can overcome some difficulties such as the lack of selectivity. Online attenuated total reflection infrared (ATR-IR) spectroscopy in conjunction with chemometrics has been proved to be a powerful choice for process analysis [19, 20].

The physical meaningful solution was obtained by classical multivariate curve resolution-alternating least squares (MCR-ALS) since using “natural” constraints. However, MCR-ALS method has a principle problem that ambiguities existed in the solution [22]. Hybrid hard- and soft-modelling multivariate curve resolution-alternating least squares (HS-MCR) analysis [23–30] has been widely used in process analysis in order to reduce the ambiguities and find a unique solution. It also provides the pure spectra and concentration profiles of principal components, in addition to the reaction rate constants from data matrices of unresolved mixture spectra recorded in synthesis systems. For HS-MCR, no prior or little information is needed about the nature and composition of the compounds involved in the processes, and the raw mixed measurement is decomposed into a small bilinear model of pure contributions that can help to interpret physical and chemical properties in dynamics of the chemical reaction system.

In this work, the aim of this study is to investigate the ability of online ATR-IR spectroscopy combined with the novel approach of HS-MCR analysis to monitor and detect changes in structural properties of compound during DAT synthesis processes. In order to obtain the number of principal components, the subspace comparison method (SCM) [31, 32] was used to analyze the IR spectra matrix. Then, the pure IR spectra and concentration profile of every substance were obtained by HS-MCR. And the extent of the rotation ambiguity was estimated from the band boundaries of feasible solutions calculated using the MCR-BANDS [33–37] procedure. The synthesis mechanism of DAT was proposed based on the changes of functional groups in IR spectra.

## 2. Materials and Methods

### 2.1. Reagents and Instruments

Cyanoguanidine (99.5% purity, Acros Organics) and hydrazine dihydrochloride (99% purity, Shanghai Meryer Chemical Technology Co., Ltd.) were commercially purchased from companies without further treatment. IR spectra were recorded with a Vertex 70 spectrometer (Bruker Optics, Ettlingen, Germany) equipped with an ATR probe (IN350-T, Germany) made of diamond combined with bundles of mid-IR optical fibers and a liquid nitrogen cooled mercury-cadmium-telluride (MCT) detector (Bruker Optics, Ettlingen, Germany) with a range of 4000–600 cm^−1^. All spectra were recorded with a 4 cm^−1^ spectral resolution. The scanning frequency of background and the sample were 16 scans per second.

### 2.2. Synthesis of DAT

Firstly, an online ATR-IR probe was inserted to a 50 mL three-necked flask which was filled with deionized water (30 mL). Secondly, the background was scanned at 20.0°C. Then, cyanoguanidine (2.50 g, 29.73 mmol) was dissolved in the deionized water and was monitored simultaneously. Hydrazine dihydrochloride (3.75 g, 35.75 mmol) was added to the above solution slowly. The above mixture was kept in 48–50°C for 90 min and then stopped to monitor. Subsequently, the pH of the mixture solution was adjusted to 10.5 with NaOH solution (3 mol/L). Finally, the crude product obtained by the vacuum evaporation of the solvent was further purified by recrystallization to obtain the desired product (2.59 g, 26.14 mmol, 88% yield).

### 2.3. IR Spectral Data

**Figure 2:**
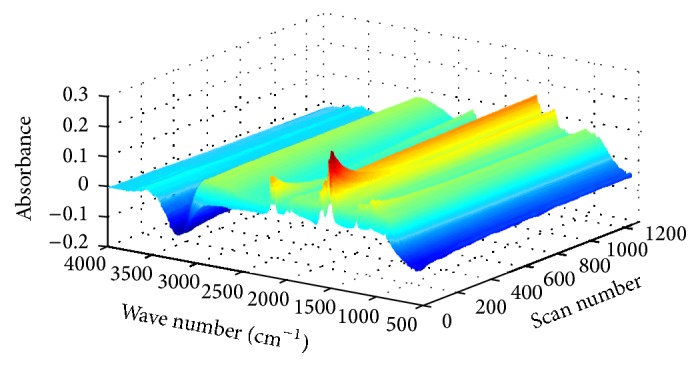
The three-dimensional diagram of online IR spectra recorded during the synthesis of DAT.

The IR spectra data were collected and shown as three-dimensional diagram (as shown in Figure 2), and as it was converted into a two-dimensional matrix **D** with the size of 1763 × 1299 by the Bruker software package OPUS, where 1299 is the number of spectra recorded of synthesis process collected in column, and 1763 is the number of wavelengths. SCM and HS-MCR were used to analyze the data matrix **D**. The MATLAB code of HS-MCR and MCR-BANDS was downloaded from the website [38] and all the calculations were carried out by MATLAB 7.4 (MathWorks, USA).

### 2.4. Hybrid Hard- and Soft-Modelling Multivariate Curve Resolution-Alternating Least Squares (HS-MCR) Method

Based on Beer–Lambert law, each chemical synthesis process monitored by ATR-IR spectroscopy gives a data matrix **D**, which can be described as follows:

(1) $$\begin{array}{c} D=CS^{T}+E. \end{array}$$

Columns of the matrix **D** are the ATR-IR spectra recorded at different reaction times, and rows of matrix **D** are the kinetic traces recorded at different wave numbers. **C** as a function of reaction time is the matrix of the concentration profiles of the resolved compounds, and **S**^*T*^ is the matrix of related resolved IR spectra of pure component. Resolved concentration profiles provide knowledge on the evolution of compounds involved in the synthesis process and the resolved spectra profiles allow compound identification. Finally, **E** is the experimental error matrix.

**Figure 3:**
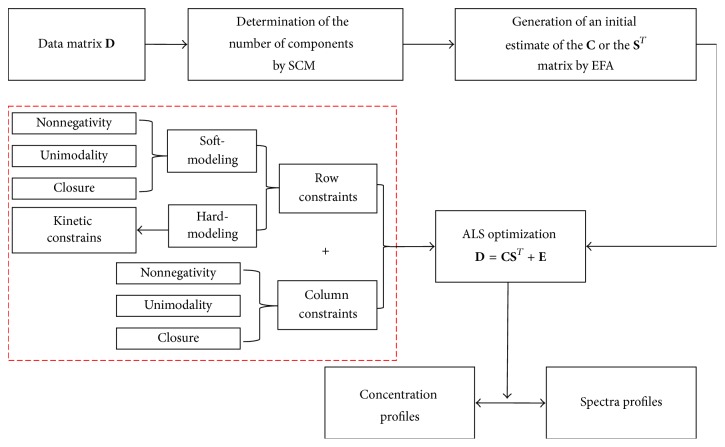
Flowchart of the HS-MCR procedure; the virtual frame represents the constraints of hard- and soft-modelling.

MCR-ALS method [25, 39, 40] targets resolving the bilinear model **D** = **CS**^*T*^ shown in (1) by using the sole information contained in the raw dataset **D**. The flowchart of this approach can be summarized in Figure 3 [25].

The resolved profiles in **C** and **S**^*T*^ may not be unique, being subject to rotational ambiguities. Therefore, constraints are used to limit the number of possible solutions and to provide chemically meaningful concentration and spectra profiles. In this work, the nonnegativity, closure and kinetic constraints were selected in the concentration direction.

The quality of the model fit can be assessed from explained variance (*R*^2^) and the lack of fit (LOF) parameter, that are defined as

(2) R2=∑i,jdij2−∑i,jeij2∑i,jdij2LOF%=100∑i,jeij2∑i,jdij2,

where *d*_*ij*_ is an element of the experimental data matrix and *e*_*ij*_ is the related residual value obtained from the difference between the experimental data matrix (**D**) and the reproduced data matrix (**S**^*T*^).

HS-MCR is a variant of MCR-ALS using hard-modelling information. HS-MCR includes physicochemical models as an additional constraint to model the shape of the concentration profiles. Thus, the selected soft-modelling concentration profiles in **C** are passed to a nonlinear fitting routine in each iteration; at the same time, these concentration profiles obey a preselected physicochemical model. There are some advantages in the HS-MCR algorithm over pure hard- or soft-modelling approaches [23, 25, 26, 28, 29, 38]. Firstly, the introduction of the hard-modelling constraint significantly decreases the rotational ambiguity linked to the classical soft-modelling approach and the reaction rate constants are provided as additional information. Secondly, the physicochemical model can be applied to some or to all the absorbing species in **C**, which is not possible in classic hard-modelling approaches. Finally, another main advantage of HS-MCR is the possibility of performing simultaneous analysis of several experiments and fitting different kinetic models to them.

## 3. Results and Discussion

### 3.1. Determination of the Number of Principal Components

The determination of the number of principal components could contribute to confirming the number of intermediates during synthesis process, which helps to deduce the reaction mechanism. Initially, the SCM [31, 32] was used to determine the chemical ranks in multivariable statistical analysis with the ability to accurately determine chemical ranks in the spectra highly similar system. In SCM procedures, base vectors of measurement matrix were extracted by two methods: principal component analysis (PCA) and simple to use interactive self-modelling mixture analysis [27], respectively. Then, one group is represented as *A* = [*a*_1_, *a*_2_,…, *a*_*N*_], and the other is represented as *B* = [*b*_1_, *b*_2_,…, *b*_*N*_] via the Gram-Schmidt process. The *i*, *j* vector in *A*, *B* is represented as *a*_*i*_, *b*_*j*_, respectively. The subspace dimension is *N*.

If we use {*b*_*j*_} as a base to present *a*_*i*_,

(3) $$\begin{array}{c} a_{i}=\overset{A}{\underset{j=1}{\sum }}b_{j}\left(\;b_{j}^{T}a_{i}\;\right)+e_{i}. \end{array}$$

The vector *e*_*i*_ presents that the missing part of *a*_*i*_ orthogonalizes {*b*_*j*_}. After square modulus operations for *a*_*i*_ were performed in the above equation, we calculated using the following equations:

(4) 1aiTai=∑j,kbiTabjTbkbkTai+eiTei=∑jaiTbj2+eiTei.

According to (4), we can get ∑_*j*_(*a*_*i*_^*T*^*b*_*j*_)^2^ < 1. If the subspace spanned by *A* belongs to the subspace spanned by *B*, it means that all the *a*_*i*_ (*i* = 1,2,…, *N*) belong to *B*; similarly, *B* can be measured by

(5) TL=∑i,jaiTbj2=Tr⁡ATBBTA,i=1,2,…,L;  j=1,2,…,L.

Tr⁡(·) represents the trace of the matrix. Obviously, 0 < *T*(*N*) < *N*. Therefore, we can define the subspace difference degree as *D*(*N*) = *N* − *T*(*N*). *D*(*N*) denotes the function of the subspace difference.

**Figure 4:**
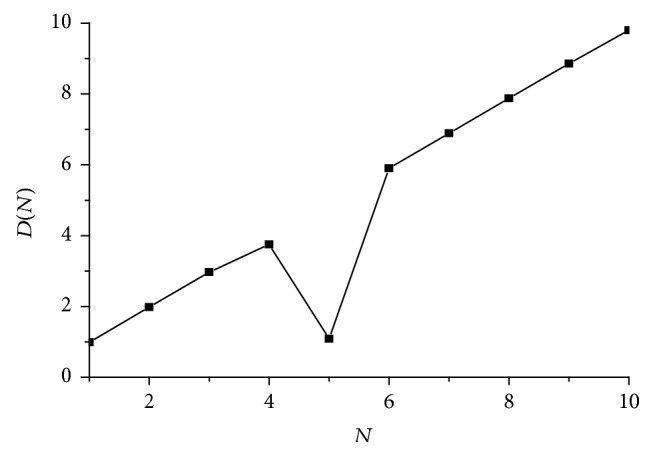
Number of principal components with subspace difference degree by the SCM.

In SCM procedures, each group of base vectors is linearly correlated. Once the unsuitable principal component number is chosen, the degree of the subspace difference would significantly increase due to the breakage of linear connection. Therefore, the appropriate principal component number appears with a minimum subspace difference degree.  Figure 4 shows the relationship between the number of principal components with subspace difference degree. It can be seen that subspace difference degree (*D*) is gradually increased with the number of principal components' increase, but when the principal components number is 5, the subspace difference degree is minimum, so 5PCs were selected and used in the subsequent work.

### 3.2. IR Spectra Resolution by Different Chemometrics and Reaction Rate Constants Determination

The pure IR spectra of 5 principal components were extracted from the matrix **D** by fast independent component analysis (ICA) [41], kernel independent component analysis (KICA) [32], mutual information least dependent component analysis (MILCA) [42], and HS-MCR algorithm, respectively. At the beginning of the calculation, the new estimates of source IR spectral matrix **S**^*T*^ were obtained through each iterative calculation until convergence. In particular, the HS-MCR calculation begins with the initial concentration estimated by evolving factor analysis (EFA) [27]. A hypothesis of three consecutive first-order kinetic models ($\text{A}+\text{B}\overset{k_{1}}{\to }\text{C}\overset{k_{2}}{\to }\text{D}\overset{k_{3}}{\to }\text{E}$) linked to the DAT formation was used to constrain the concentration of the HS-MCR procedure.

**Figure 5:**
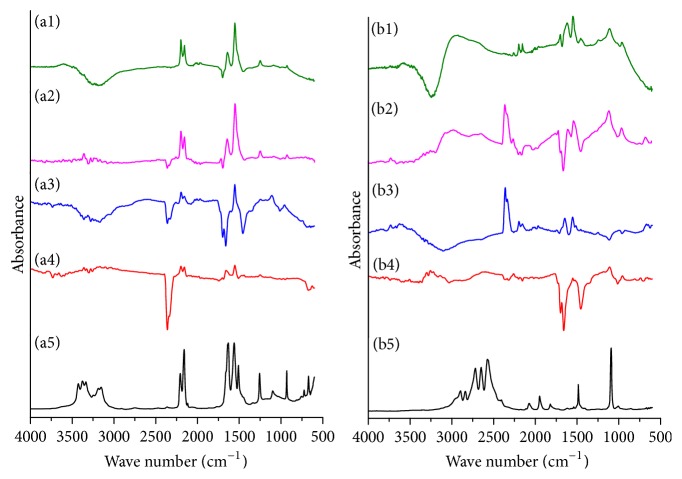
IR spectra of cyanoguanidine ((a1): HS-MCR; (a2): MILCA; (a3): KICA; (a4): fast ICA; (a5): ATR probe) and hydrazine dihydrochloride ((b1): HS-MCR; (b2): MILCA; (b3): KICA; (b4): fast ICA; (b5): ATR probe) obtained from different chemometrics.

The IR spectra obtained by different advanced chemometrics algorithm and the actual spectra of cyanoguanidine and hydrazine dihydrochloride solution measured by ATR probe were shown in Figure 5. As can be seen in Figure 5, some of the shapes of the characteristic absorption peaks were similar. But compared with the real spectra, the shapes of some characteristic absorption peaks obtained by three ICA (fast ICA, KICA, and MILCA) algorithms were reversed, especially for the C≡N bond (2200 cm^−1^, 2154 cm^−1^) and C=N bond (1647 cm^−1^, 1554 cm^−1^) of cyanoguanidine and the principal absorption peaks of hydrazine dihydrochloride (2490 cm^−1^, 1550 cm^−1^). But the plot with coincided real and resolution by HS-MCR spectra absorbance data that has been represented in Figure 5, which is a strong evidence for high quality of fitting result. In addition, HS-MCR analysis not only helps to minimize the ambiguity associated with the soft-modelling data decomposition but also provides estimates of the process parameters as additional information such as reaction rate constants. Thus, HS-MCR analysis was selected to deduce the synthesis mechanism of DAT.

**Figure 6:**
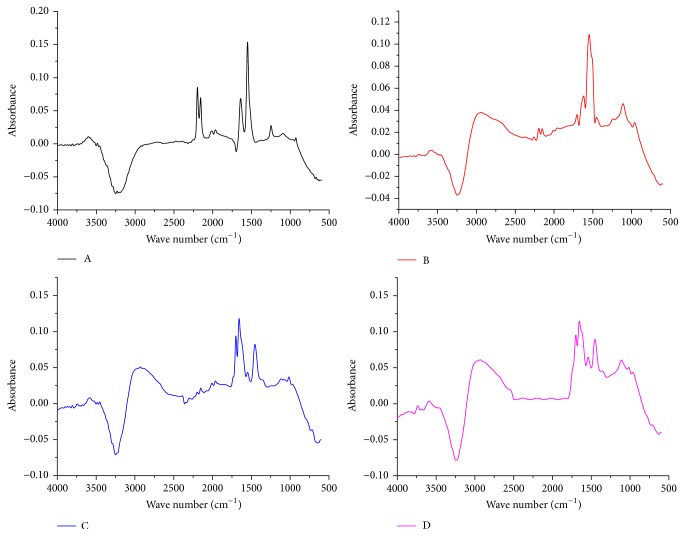
IR spectra were analyzed by HS-MCR (A: cyanoguanidine; B: intermediate 1; C: intermediate 2; D: DAT).

**Figure 7:**
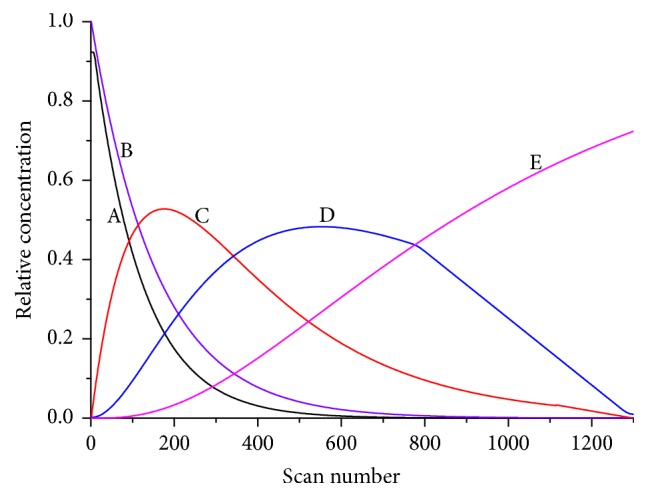
Concentration profiles were analyzed by HS-MCR (A: cyanoguanidine; B: hydrazine dihydrochloride; C: intermediate 1; D: intermediate 2; E: DAT).

The IR spectra and the concentration profile from each principal component obtained by HS-MCR analysis were shown in Figures 6 and 7, respectively. At the same time, according to the kinetic mode, the reaction rate constants were acquired (*k*_1_ = 0.003874 s^−1^, *k*_2_ = 0.0004358 s^−1^, *k*_3_ = 0.0006246 s^−1^). The explained variance *R*^2^ = 0.9976 and the lack of fit parameter LOF% = 0.4846 were obtained simultaneously. It can be seen that the value of *R*^2^ approach to 1 and LOF% is similar to 0; that preliminary indicates the high quality of the model fit. In the concentration profile, it shows there has two intermediates in the synthesis process. Moreover, the order of magnitude for *k*_2_ agrees with *k*_3_, and they are lower than *k*_1_ an order of magnitude.

### 3.3. The Extent Study of Rotational Ambiguities by MCR-BANDS

In the ideal state, the HS-MCR method was used to assist in obtaining reliable results which were in agreement with experimental observations. However, in practice, the solution of HS-MCR has the rotational ambiguities. In order to evaluate the effect of rotation ambiguities associated with a particular MCR solution and to measure its extent, the area of feasible solutions (AFS) [43] is computed using FAC-PACK toolbox [44] with polygon inflation algorithm or MCR-BANDS method [31–35, 45] which based on the relative signal component contribution function (SCCF) [33, 46] of each component. The MCR-BANDS method provides an easy and flexible estimation of the extension of rotation ambiguities for any number of components and for all types of constraints. And the extension of rotation ambiguities estimated by the MCR-BANDS method and the AFS calculated by FAC-PACK are in good agreement [47]. Therefore, the MCR-BANDS method was applied in the data matrix **D** to evaluate the existence of rotational ambiguities which were described by HS-MCR. The constraints used in HS-MCR were similar to MCR-BANDS. The method was based on the calculation of the relative contribution of every component in a mixture, the equation was as follows:

(6) $$\begin{array}{c} f_{n}=\frac{\left\|c_{n}s_{n}^{T}\right\|}{\left\|CS^{T}\right\|}, \end{array}$$

where *f*_*n*_ is a scalar value which gives the relative signal contribution of a particular component to the whole signal for the mixture of *N* components (*n* = 1,…, *N*). This relative signal contribution is measured by the quotient of two norms (Frobenius norm), the one from the signal of the considered component *n*, ‖**c**_*n*_**s**_*n*_^*T*^‖, and the one from the whole signal considering all components of the system together, ‖**CS**^*T*^‖.

The obtained maximum and minimum values of the relative contribution function (*f*^max^ and *f*^min^, resp.) were listed in Table 1. It can be seen that a certain amount of ambiguity is found in 5 principal components. The extent of ambiguity is estimated by the *f*^max^ − *f*^min^ value, which varies from 0.1356 to 0.5640 depending on the principal components; they are approached to 0. There is no difference between the *f*^max^ and *f*^min^ values for all the resolved components. It means that there is no remaining rotation ambiguity. The elimination of the rotational ambiguity could be attributed to the application of appropriate constraints. To avoid rotational ambiguity in MCR methods, besides the “natural” constraints, the hard-modelling information constraints were used. It seems that the hard modelling in the iterative procedure leads to a unique solution. The value of *f*^max^ − *f*^min^ further verifies the high quality of the model fit and the unique solution.

### 3.4. Synthesis Mechanism of DAT

**Figure 8:**
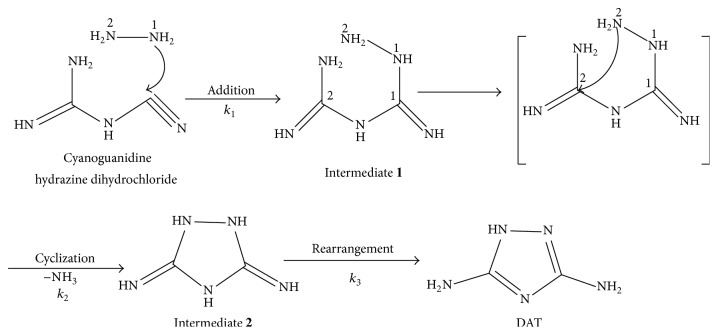
Synthesis mechanism of DAT.

Based on analysis of the IR spectra of reactants, intermediates, and product (Figure 6), the synthesis mechanism of DAT is proposed in Figure 8. The reaction is initiated by the addition of hydrazine dihydrochloride to the cyanoguanidine solution. Figure 6A shows the IR spectra of cyanoguanidine and Figure 6B shows the IR spectra of intermediate** 1**; they were obtained by HS-MCR method. It can be seen that the intensity of absorption peaks of C≡N bond (2200 cm^−1^, 2154 cm^−1^) from intermediate** 1** is weaker than that from cyanoguanidine, while the peaks intensities of C=N bond (1647 cm^−1^, 1564 cm^−1^) and C−N bonds (1350–1100 cm^−1^) from intermediate** 1** are higher than those from cyanoguanidine. The reason is that the C≡N bond of cyanoguanidine is attacked by N1 atoms of hydrazine dihydrochloride under acidic conditions, and this leads to the cleavage of C≡N bond of cyanoguanidine and the formation of a new C=N and C–N bonds. The –NH–NH_2_ group is added to the C atoms from C≡N bond of cyanoguanidine simultaneously to generate intermediate** 1**.

It can be seen from Figures 6B and 6C that the absorption peak of C≡N bond (2200 cm^−1^, 2154 cm^−1^) from intermediate** 2** is further reduced than that from intermediate** 1**, and a skeleton vibration (1660–1450 cm^−1^) of heterocyclic compounds is preliminarily formed. It illustrates that the C atom of C=N bond is attacked by the N2 with the reaction continuing. Subsequently, an unstable intermediate** 1** is collapsed via the cleavage of C–N bond at C2, and a new heterocycle is formed to generate intermediate** 2**.

Finally, Figure 6D shows the absorption peak of C≡N bond (2200 cm^−1^, 2154 cm^−1^) of the product disappeared, which confirms that C≡N bond does not exist in product, and the absorption peak of N=C–C=N group (1600 cm^−1^) is formed. It suggests that the unstable intermediate** 2** has been transformed to final product through the rearrangement reaction.

## 4. Conclusion

In this study, online ATR-IR spectroscopy combined with HS-MCR analysis was used to investigate the synthesis process of DAT from cyanoguanidine and hydrazine dihydrochloride. The SCM was used to analyze the IR spectroscopy matrix, and 5 principal components were acquired. The effective spectral information of the principal components in chemical synthesis reactions was extracted from mixed matrix by the HS-MCR algorithm, and the reaction rate constants of DAT formation reaction were obtained. The extent of the rotation ambiguity was estimated from the band boundaries of feasible solutions calculated by the MCR-BANDS. The explained variance *R*^2^ = 0.9976 and the lack of fit parameter LOF% = 0.4846 are obtained by HS-MCR, and the *f*^max^ − *f*^min^ value varies from 0.1356 to 0.5640 obtained by MCR-BANDS, that indicate the high quality of the model fit by HS-MCR algorithm. The synthesis mechanism of DAT was deduced through the changes of functional groups in IR spectra. The advantages of HS-MCR analysis are the ability to examine the complete IR spectrum, resolve overlapping bands, and obtain the reaction rate constants. This development demonstrated that online ATR-IR combined with HS-MCR method can be applied to study the chemical synthesis mechanism and provide a strong technical support for the research and development of process analytical technology.

## Figures and Tables

**Table 1:** The maximum and minimum values of rotation ambiguity which was measured by components relative contribution function.

	*f* ^max^	*f* ^min^	*f* ^max−min^
1st component	0.1916	0.01802	0.1736
2ed component	0.1530	0.01723	0.1356
3rd component	0.4720	0.01710	0.4549
4th component	0.5820	0.01803	0.5640
5th component	0.3060	0.01711	0.2889
